# Comparison of Postoperative Outcomes of Sutureless *versus* Stented Bioprosthetic Aortic Valve Replacement

**DOI:** 10.21470/1678-9741-2020-0404

**Published:** 2022

**Authors:** Yesim Guner, Ayse Çiçek, Mehmet Karacalilar, Burak Ersoy, Mugisha Kyaruzi, Burak Onan

**Affiliations:** 1 Department of Cardiovascular Surgery, University of Health Sciences Turkey, Istanbul Mehmet Akif Ersoy Thoracic and Cardiovascular Surgery Hospital, Istanbul, Turkey.

**Keywords:** Aortic Valve, Cardiopulmonary Bypass, Heart Valve Prosthesis, Bioprosthesis, Echocardiography, Intensive Care Units

## Abstract

**Objective:**

Sutureless aortic valve replacement (Su-AVR) offers an alternative to supra-annular stented biological aortic prostheses. This single-center study aimed to compare early outcomes after aortic valve replacement with sutureless and conventional stented bioprostheses.

**Methods:**

In this retrospective study, we analyzed 52 patients who underwent aortic valve replacement with sutureless and stented bioprostheses between January 2013 and October 2017. Sorin Perceval S sutureless valves were implanted in group 1 and Sorin Mitroflow stented bioprosthetic valves were used in group 2. Postoperative outcomes, including demographics, cardiopulmonary bypass (CPB) times, cross-clamp times, morbidity and mortality, as well as echocardiography in the first month, were compared.

**Results:**

Mortality occurred in 1 (3.6%) patient in group 1, and in 2 (8.3%) patients in group 2 (*P*=0.186). Group 1 had significantly shorter CPB (61.6±26.1 min *vs*. 106.3±32.7 min, *P*=0.001) and cross-clamp (30.9±13.6 min *vs*. 73.3±17.3 min, *P*=0.001) times. The length of stay in the intensive care unit (1.9±1.3 days *vs*. 2.4±4.9 days, *P*=0.598) and hospital stay (7.6±2.7 days *vs*. 7.3±2.6 days, *P*=0.66) were similar. Postoperatively, there was no statistically significant difference between the two groups in echocardiography results, and morbidities. The mean aortic valve gradient was 13.5±5.8 mmHg in group 1 and 14.5±8.0 mmHg in group 2 (*P*=0.634). Paravalvular regurgitation was diagnosed in 3 (10.7%) patients in group 1 and in 1 (4.2%) patient in group 2 (*P*=0.220).

**Conclusions:**

Su-AVR resulted in shorter cross-clamp and CPB times. However, early mortality, postoperative morbidity, and echocardiography results were similar between groups.

**Table:** 

Abbreviations, acronyms & symbols	
AS	= Aortic valve stenosis
AVR	= Aortic valve replacement
c-AVR	= Conventional aortic valve replacement
CPB	= Cardiopulmonary bypass
PLT	= Platelet
TEE	= Transesophageal echocardiography
SPSS	= Statistical Package for the Social Sciences
Su-AVR	= Sutureless aortic valve replacement

## INTRODUCTION

Aortic valve stenosis (AS) has been the most common valvular heart disease among elderly patients, with a prevalence of approximately 3% ^[1]^. Surgical aortic valve replacement is the gold standard treatment for severe symptomatic AS ^[1-3]^. However, there has been a growing interest in the use of sutureless bioprostheses to decrease operative times and associated postoperative complications. Sutureless aortic bioprostheses offer an alternative for patients who are eligible for surgical aortic valve replacement. Nevertheless, supra-annular aortic biological prostheses have been widely used during the last decades. These valves increase the flow relative to the annular area of the aortic valve compared to the intra-annular position in some of the other bioprostheses. Especially in patients with small aortic annulus (19 and 21 mm), they are considered ideal prosthesis in elderly patients ^[4]^. It has been reported that the absence of structural valve deterioration in pericardial aortic valves is above 90% ^[5]^. According to the current literature, sutureless valves provide superior hemodynamic outcomes with reduced gradients and reduced aortic cross-clamp and cardiopulmonary bypass (CPB) times compared to conventional aortic valve replacement (c-AVR) ^[2-12]^. But most evidence regarding sutureless aortic valve replacement is limited to observational studies. Only one small randomized controlled study has demonstrated its feasibility, safety, and efficacy ^[11]^. There are still limited data on the outcomes of biological and sutureless aortic prosthesis. In this study, we aimed to compare the early outcomes after aortic valve replacement with sutureless bioprostheses and conventional stented bioprostheses. 

## METHODS

### Patients

After approval from the hospital ethics committee, we retrospectively reviewed 52 patients who underwent aortic valve replacement due to severe aortic stenosis using sutureless (n=28) and stented aortic bioprostheses (n=24) between January 2013 and October 2017. Patients who underwent isolated aortic valve replacement due to severe symptomatic aortic stenosis using biological valves were included. Sorin Perceval S sutureless valves (Sorin, part of LivaNova PLC) were implanted in group 1. Sorin Mitroflow stented bioprosthetic valves (Sorin Group, Milan, Italy) were implanted in group 2. Patients who underwent reoperation and other combined surgeries such as coronary artery bypass grafting and ascending aorta replacement were excluded. All operations were performed by the same surgical team. The patients were evaluated by results of preoperative characteristics and perioperative data, including CPB and cross-clamp times, postoperative morbidity and mortality, and echocardiography in the first month.

### Surgical Technique

After general anesthesia, all surgeries were performed via full sternotomy. Cannulation was performed through the ascending aorta and the right atrium. Following CPB initiation, the ascending aorta was cross-clamped and a transverse aortotomy incision was performed. Isothermic blood cardioplegia was delivered to allow diastolic cardiac arrest at 30 °C. Maintenance doses were given to each coronary ostium every 20 minutes. However, taking into consideration the valve height in patients in which we planned to use a sutureless valve, aortotomy was performed a little more distally over the sinotubular junction (about 1 cm above the sinotubular junction). The valve leaflets were resected and decalcified to prevent paravalvular leak in both groups. 

### Sutureless Valve Implantation

**Fig. 1:**
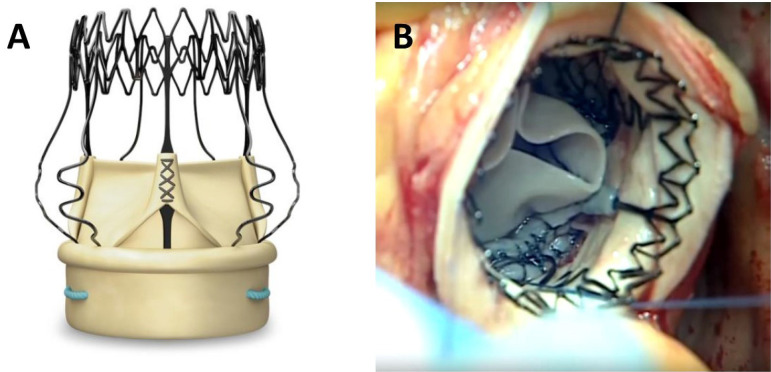
Sutureless aortic valve (A) and operative view after implantation (B).

Sorin Perceval S sutureless aortic valve (Sorin Biomedica Cardio Srl, Saluggia, Italy) was used in group 1 (Figure 1). The valve was sized according to the annular diameter of the aortic valve, with a gentle passage through the valve orifice. After crimping procedure of properly sized aortic sutureless valve, the valve was washed with saline solution. The delivery system was loaded with the collapsed stent-mounted valve and guided to its correct position by sliding it over three guiding sutures (4-0 polypropylene), positioned at the nadir level of each resected cusp. Once the delivery system was in place, the prosthesis was deployed, the guiding sutures were removed and the valve was put in place; at this point, post-dilation modeling was performed with a dedicated balloon (30 seconds at a pressure of 4 atmospheres). The valve was washed with 37 °C saline solution, making it easier for the nitinol ring to expand. 

### Surgical AVR with Bioprosthetic Valves

Sorin Mitroflow bioprosthetic valve (Sorin Group, Inc., Milan, Italy) was used in group 2. After sizing the proper valves, washing procedures with saline solution were done. Pledgets 2-0 Politer sutures were passed through the annulus with the pledgets located subannulary. After all the sutures were passed symmetrically through the valve ring, the valve was placed in a supra-annular position. Transesophageal echocardiography (TEE) was performed during the procedure to evaluate valve opening, position, presence of paravalvular and valvular leak in both groups. Then, aortotomy was closed in a double-layered fashion. Following implantation of the valves, chest tubes and temporary epicardial pacing wires were placed. Hemostasis was done and the sternum was closed traditionally. 

### Postoperative Follow-Up

Patients were transferred to the intensive care unit and extubated. They were transferred to the ward on postoperative day 1, if there was no need for further follow-up in the intensive care unit. Postoperative ventilation time, intensive care unit stay, and the length of hospital stay were reviewed. Patients were followed-up for major adverse events, such as cardiac, neurological, pulmonary, and other organ dysfunctions. Mortality and other morbidities were recorded. All patients underwent intraoperative and postoperative echocardiography examinations to evaluate the prosthetic valve and ventricular functions. 

### Statistical Analysis

Statistical analysis of the study was done with SPSS 17.0 for Windows. In the analysis, first, the normal distributions of the data were examined. For this, the one-sample Kolmogorov-Smirnov test was applied. Data showed normal distribution (*P*>0.05) and the main analyzes were carried out. Frequency distributions (number, percentage, mean and standard deviation) of the data were made and then independent samples t-test (Student’s t-test) was applied for statistical significance. Significance levels were taken as 95%. Those with a *P*<0.05 were considered statistically significant. 

## RESULTS

Preoperative characteristics of the patient groups are summarized in Table 1. Both groups were similar in terms of demographic data, functional class, EuroSCORE II values (3.2±1.6 *vs*. 3.2±2.5, *P*=0.93) and comorbidities. Patients presented with good ventricular functions and severe gradient through the aortic valve (mean: 51.4±10 mmHg *vs*. 58.4±15.2 mmHg, *P*=0.05). 

**Table 1:** Preoperative demographic data.

Variable		Su-AVR group	c-AVR group	*P*-value
Age (years)		73±6.8	72.9±4.3	0.94
Male sex		14 (50)	19 (79)	0.24
Body surface area (m^2^)		1.8±0.2	1.9±0.2	0.20
NYHA class	Class 2	18 (64.3)	18 (75.0)	0.45
	Class 3	10 (35.7)	6 (25.0)	0.34
EuroSCORE II		3.2±1.6	3.2±2.5	0.93
Obstructive lung disease		16 (57)	17 (70)	0.31
Hypertension		16 (57)	12 (50)	0.61
Diabetes mellitus		7 (25)	9 (38)	0.34
Peripheral artery disease		6 (21)	4 (17)	0.47
Cerebrovascular event		4 (14)	2 (8)	0.51
Coronary artery disease		7 (25)	10 (42)	0.20
Chronic atrial fibrillation		3 (10.7)	3 (12.5)	0.52
Echocardiography results	Left ventricular ejection fraction (%)	56.8±9.1	60.6±5.1	0.07
	Maximum aortic gradient (mmHg)	82.4±11.9	94±22.8	0.07
	Mean aortic gradient (mmHg)	51.4±10	58.4±15.2	0.05
	Left ventricular end-diastolic diameter (mm)	46.7±6.9	49.1±6.5	0.21
	Interventricular septum (mm)	12.9±1.8	13.5±2.1	0.23
	Posterior wall (mm)	12±1.3	12.7±1.4	0.09

Continuous data were presented as mean±standard deviation and categoric data were presented as numbers (%). A *P*-value <0.05 was considered significant. c-AVR=conventional aortic valve replacement; Su-AVR=sutureless aortic valve replacement

Table 2 shows the distribution of implanted valve size. In the c-AVR group, 12 (50.0%) patients had a 21-mm prosthesis and 7 (29.2%) patients had a 23-mm prosthesis. In the Su-AVR group, patients had medium (39.3%), large (28.6%) and X-large (28.6%) prostheses. 

**Table 2:** Distribution of implanted prosthetic aortic valve sizes.

Variable		Su-AVR group	c-AVR group
Sorin Mitroflow bioprostheses	No 19	-	1 (4.2)
	No 21	-	12 (50.0)
	No 23	-	7 (29.2)
	No 25	-	3 (12.5)
	No 27	-	1 (4.2)
Sorin Perceval sutureless valve/annular diameter	Small - 19-21 mm	1 (3.6)	-
	Medium - 21-23 mm	11 (39.3)	-
	Large - 23-25 mm	8 (28.6)	-
	X-large - 25-27 mm	8 (28.6)	-

Data were presented as numbers (%).

The operative and early postoperative outcomes were presented in Table 3. Mortality occurred in 1 (3.6%) patient in the Su-AVR group and in 2 (8.3%) patients in the c-AVR group (*P*=0.186). CPB (61.6±26.1 *vs*. 106.3±32.7 min, *P*=0.001) and cross-clamp (30.9±13.6 *vs*. 73.4±17.3 min, *P*=0.001) times were significantly shorter in the Su-AVR group. Mean length of intensive care unit stay and hospital stay were similar between groups. Postoperative morbidities showed similarity. There was no need for reoperation. 

**Table 3:** Operative and postoperative outcomes.

Variable	Su-AVR group	c-AVR group	
Mortality	1 (3.6)	2 (8.3)	0.186
Cardiopulmonary bypass time (min)	61.6±26.2	106.3±32.7	0.001
Cross-clamp time (min)	30.9±13.6	73.4±17.3	0.001
Ventilation time (hours)	8.6±3.4	13.4±17.9	0.216
Intensive care unit stay (days)	1.9±1.3	2.4±4.9	0.598
Length of hospital stay (days)	7.6±2.7	7.3±2.6	0.669
Platelet count	79.2±40.2	102.7±55.3	0.083
Use of inotropic support	13 (46)	8 (33)	0.413
Prolonged inotropic support (>24 hours)	3 (10.7)	2 (8.3)	0.382
New-onset atrial fibrillation	3 (10.7)	3 (13.0)	0.898
Re-exploration for bleeding	1 (3.6)	1 (4.2)	0.164
Permanent pacemaker implantation	2 (7.0)	-	
Sepsis	-	2 (8.3)	
Transient ischemic attack	1 (3.6)	-	
Reoperation	-	-	

Continuous data were presented as mean±standard deviation and categoric data were presented as numbers (%). A *P*-value <0.05 was considered significant. c-AVR=conventional aortic valve replacement; Su-AVR=sutureless aortic valve replacement

Postoperative echocardiography results were presented in Table 4. Mean postoperative aortic gradients were 13.5±5.8 mmHg in the Su-AVR group and 14.5±8.0 mmHg in the c-AVR group (*P*=0.634). Central aortic regurgitation was diagnosed in 3 (10.7%) patients in the Su-AVR group and in 1 patient in the other group (*P*=0.224). Paravalvular regurgitation was found in 3 (10.7%) patients in the Su-AVR group and in 1 (4.2%) patient in the c-AVR group (*P*=0.220). Paravalvular regurgitation was mild in 2 patients and moderate in 1 patient in the Su-AVR group. 

**Table 4:** Postoperative echocardiography results.

Echocardiography results	Su-AVR group	c-AVR group	*P*-value
Left ventricular ejection fraction (%)	56.5±8.1	58.3±6.3	0.411
Maximum aortic gradient (mmHg)	25.7±10.4	25.9±13.8	0.960
Mean aortic gradient (mmHg)	13.5±5.8	14.5±8.0	0.634
Central aortic regurgitation	3 (10.7)	1 (4.2)	0.224
1+	2 (7.1)	1 (4.2)	
2+	1 (3.6)	-	
Paravalvular aortic regurgitation	3 (10.7)	1 (4.2)	0.220
1+	2 (7.1)	1 (4.2)	
2+	1 (3.6)	-	
Left ventricular end-diastolic diameter (mm)	47.1±5.5	50±5.5	0.078
Left ventricular end-systolic diameter (mm)	32.4±8.4	32.5±6.1	0.960
Interventricular septum (mm)	12.2±1.7	12.7±1.3	0.295
Posterior wall (mm)	11.5±1.5	12.1±1.2	0.162

Data were presented as mean±standard deviation. A P-value <0.05 was considered significant. c-AVR=conventional aortic valve replacement; Su-AVR=sutureless aortic valve replacement

## DISCUSSION

Sutureless valves have recently gained popularity as they reduce the operation time and facilitate minimally invasive surgery in high-risk patients ^[2,3]^. Due to the higher cost and limitations of transcatheter aortic valve implantation procedure, sutureless valves have become a remarkable option, especially in the elderly and in the high-risk population. In this study, we compared the early outcomes of sutureless valves and stent bioprostheses for aortic valve replacement. Although results showed a significant decrease in operative times with sutureless valves, there was no difference in postoperative outcomes regarding mortality, complications, and echocardiographic results. Our experience revealed that sutureless valves could be advantageous in patients with small aortic root. 

The use of sutureless valves can be recommended in patients with advanced age, previous cardiac operations, concomitant procedures, calcified homograft, porcelain aorta, and small aortic root ^[2,3]^. These valves can also be useful in minimally invasive surgery via right anterior small thoracotomy or J-sternotomy procedures. Technically, transverse aortotomy should be performed well above the sinotubular junction in sutureless valve implantation, whereas traditional oblique aortotomy is done for aortic valve replacement (AVR) using stented-biological aortic prostheses. Previously, Gode et al. ^[2]^ and Hanedan et al. ^[3]^ reported the safety of these procedures. 

Prolonged CPB and cross-clamp times are known as independent risk factors for postoperative morbidity and mortality in cardiac surgery ^[13,14]^. In many studies, CPB and cross-clamp times were found to be lower in sutureless valves compared to conventional AVR, as no time was spent on stitching and knotting. Flameng et al. ^[6]^ found average CPB and cross-clamp times of 46 and 20 minutes, respectively. According to the study conducted in the STS database, CPB and cross-clamp times were found to be 106 and 78 minutes, respectively, in isolated sutureless aortic valve replacement via median sternotomy ^[7]^. In the study by Smith et al. ^[8]^ to compare conventional AVR with sutureless aortic valve replacement, the authors found that the CPB and cross-clamp times in sutureless aortic valve replacement were approximately 95 and 71 minutes, respectively. This indicates that sutureless valves shorten CPB and cross-clamp times. Our study was equivalent to other studies with CPB and cross-clamp times of 61 and 30 minutes, respectively. CPB and cross-clamp times were significantly shorter in sutureless valves compared to conventional AVR.

Many studies argue that sutureless valve reduces operation time, decrease mortality and morbidity, and improves quality of life ^[15,16]^. Our study has revealed an equivalent 30-day mortality rate of sutureless valves with these studies and meta-analyzes. However, there was no significant difference in mortality with the conventional valve replacement group. A single-center large-scaled study by Gilmanov et al. ^[17]^ revealed that sutureless valve reduces mortality compared to traditional aortic valve replacement. Folliguet et al. ^[12]^ is the only known large prospective, multicentered study of sutureless valves. This 4-year follow-up study consisted of 208 high-risk patients who had undergone Perceval S implantation in which hospital mortality and 1-year mortality observed were 2.4% and 12.9%, respectively. These results can be comparable with the c-AVR group results. 

In the present study, no significant outcome difference was found between the two groups in terms of intensive care unit stay, hospital stay, postoperative drainage amount, mechanical ventilation time and postoperative morbidities. However, two patients needed permanent pacemaker implantation after sutureless valve replacement. A study performed in Germany by Pollari et al. ^[18]^ reported a shorter operation time in sutureless aortic valve replacement compared to conventional AVR. In addition, the authors reported a decreased blood transfusion, postoperative atrial fibrillation rate, mechanical ventilation time, duration of intensive care and total cost. Gilmanov et al. ^[17]^ reported shorter mechanical ventilation time in the minimally invasive approach of sutureless aortic valve replacement compared to stented bioprostheses. 

Sutureless aortic valves provides another superiority in terms of hemodynamic performance in patients who undergo aortic valve replacement due to aortic stenosis. Many studies have found that sutureless valves decrease average and maximum gradient. It also increases transvalvular flow and effective orifice area. Sadowski et al. ^[9]^ reported mean and maximum gradients of 11.6 and 6.8 mmHg, respectively, at discharge. Minh et al. ^[10]^ in their study on sutureless valves found the mean gradient of 11.1±4.6 mmHg. A multicenter, randomized study by Borger et al. ^[11]^ revealed lower gradients of sutureless valves compared to stented bioprostheses (8.5 mmHg *vs*. 10.3 mmHg). Our study has revealed the mean gradient of 13.5 mmHg in sutureless valves and there was no significant difference found between sutureless valves and stented bioprostheses.

During sutureless aortic valve replacement procedure, size measurement and proper annular decalcification are the most critical stages of the operation. Valves that are not properly sized can lead to several problems. Valves smaller than the annulus can cause paravalvular leaks, central aortic regurgitation, malposition, and migration of the valve. Valves larger than the annulus can cause excessive shear stress or even rupture of the aortic wall. They can also result in stent intussusception, hemorrhage, fatal arrhythmia, regurgitation, or hemodynamic changes in the valve. Intraoperative TEE control is essential to prevent paravalvular leaks. Many studies have reported a low rate of paravalvular leak and particularly good hemodynamic performance in Perceval valves ^[3-12]^. Our study has revealed a mild to moderate paravalvular leak in 10.7% of our patients who underwent sutureless valve replacement, but no statistically significant difference was found compared with c-AVR. The incidence of paravalvular regurgitation with stented bioprostheses was lower in the c-AVR group. 

Albacker et al. ^[19]^ reported transient postoperative thrombocytopenia in sutureless valve operation more than traditional bioprosthetic valves. Flameng et al. ^[6]^ revealed that the number of platelets decreased in the one-year follow-up after Perceval S implantation. Some studies argue that postoperative thrombocytopenia is due to transient toxic effect of Perceval S sutureless valve on platelets. Other studies claim that microhemodynamic effects of prosthetic structure may be the cause of postoperative thrombocytopenia ^[20]^. Edwards Intuity and Perceval S valves were compared in terms of postoperative thrombocytopenia in a study conducted in Italy ^[21]^. This study concluded that Perceval S implantation was accepted as an independent risk factor for the development of early postoperative thrombocytopenia. Patients who were replaced with the Edwards Intuity valve did not develop significant thrombocytopenia compared to patients who were replaced with the Perceval S valve during the early postoperative period. However, platelet level returned to its preoperative level in both groups after one year of follow-up. Authors argue that the thrombocytopenia seen in Perceval S valve during early postoperative period develops because the nitinol stent in the valve structure is not covered with any substance ^[21]^. In our study, there was no significant difference in comparison of lowest platelet (PLT) values in the postoperative period with PLT values at the discharge in both groups.

### Limitations

Retrospective design of the study protocol, limited number of patients in both groups and the lack of long-term results are major limitations. Cost analysis and analysis of long-term reintervention rate may improve the results of these interventions. Thus, further studies are still needed. 

## CONCLUSION

The results of this study showed comparable early postoperative outcomes of sutureless valves versus stented bioprosthetic aortic valves. Sutureless valves can be preferred to decrease operating time in patients referred for concomitant procedures.

**Table:** 

Authors' roles & responsibilities	
YG	Substantial contributions to the conception or design of the work; or the acquisition, analysis, or interpretation of data for the work; final approval of the version to be published
AÇ	Substantial contributions to the conception or design of the work; or the acquisition, analysis, or interpretation of data for the work; drafting the work or revising it critically for important intellectual content; final approval of the version to be published
MK	Substantial contributions to the conception or design of the work; or the acquisition, analysis, or interpretation of data for the work; drafting the work or revising it critically for important intellectual content; final approval of the version to be published
BE	Substantial contributions to the conception or design of the work; or the acquisition, analysis, or interpretation of data for the work; drafting the work or revising it critically for important intellectual content; final approval of the version to be published
MK	Substantial contributions to the conception or design of the work; or the acquisition, analysis, or interpretation of data for the work; drafting the work or revising it critically for important intellectual content; final approval of the version to be published
BO	Final approval of the version to be published
